# Efficient Isolation and In Situ Identification of Viable Circulating Tumor Cells Using Dual‐Responsive Fluorescent‐Magnetic Nanoparticles

**DOI:** 10.1002/smsc.202200061

**Published:** 2022-10-27

**Authors:** Yuwei Zhou, Xiaoshan Wang, Zhouying Luo, Xia Liu, Jianwen Hou, Shaobing Zhou

**Affiliations:** ^1^ Key Laboratory of Advanced Technologies of Materials Ministry of Education School of Materials Science and Engineering Southwest Jiaotong University Chengdu 610031 China; ^2^ Cancer Center Hospital of The University of Electronic Science and Technology of China and Sichuan Provincial People's Hospital School of Medicine University of Electronic Science and Technology of China Chengdu 610072 China; ^3^ School of Life Science and Engineering Southwest Jiaotong University Chengdu 610031 China

**Keywords:** broad-spectrum capture, circulating tumor cells, core–shell structures, in situ labeling, stimulus-responsiveness

## Abstract

Detecting circulating tumor cells (CTCs) from the peripheral blood of cancer patients is a promising approach for diagnosing early‐stage cancers, monitoring disease progression, and prescribing personalized anticancer therapy. However, it is still challenging to achieve effective and cell‐friendly isolation of CTCs due to their scarcity, heterogeneity, and vulnerability. Herein, a novel multifunctional platform based on fluorescent‐magnetic Fe_3_O_4_/Rhm B@ZIF‐8‐pTA nanoparticles (FR@Z‐pTA NPs) is developed for efficient CTCs isolation. FR@Z‐pTA NPs not only capture more than 88% of rare cancer cells for both EpCAM‐positive cells (MCF‐7, HepG2) and EpCAM‐negative cells (MDA‐MB‐231, HeLa) but also effectively release the captured cells with high efficiency (>80%) and viabilities (>90%) under cell‐friendly pH/ATP stimuli. More importantly, FR@Z‐pTA NPs exhibit excellent resistance to nonspecific adhesion of white blood cells (WBCs) and high detection sensitivity toward cancer cells in the patients’ blood. The present multifunctional CTCs detection platform integrating high sensitivity, broad‐spectrum capture, in situ fluorescent identification and cell‐friendly release offers a good solution to address current challenges of CTCs isolation from clinical blood samples.

## Graphical abstract

Heterogeneous circulating tumor cells (CTCs) with varied expression levels of epithelial cell adhesion molecule (EpCAM) are efficiently captured (>88%) and in situ identified by fluorescent‐magnetic Fe_3_O_4_/Rhm B@ZIF‐8‐pTA nanoparticles (FR@Z‐pTA NPs). Furthermore, FR@Z‐pTA NPs can gently release the captured cells with high efficiency (>80%) and viabilities (>90%) under cell‐friendly pH/ATP stimuli.

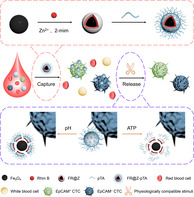

## 1 Introduction

In recent years, cancer has become a major source of mortality around the world and tumor metastasis accounts for over 90% of cancer deaths. Circulating tumor cells (CTCs), which are shed from the primary tumor sites into the peripheral blood, play a key role in forming new metastatic lesions at distant tissues and organs.^[^ ^1^, ^2^, ^3^, ^4^ ^]^ Studies have shown that using CTCs as an effective target of “liquid biopsy” is of great significance in diagnosing early‐stage cancers, monitoring disease progression, and prescribing personalized anticancer therapy.^[^ ^5^, ^6^, ^7^ ^]^ During the last decades, a series of CTC isolation methods, which are mainly based on the distinct physical properties (size, density, deformability, etc.) or unique surface biomarkers (epithelial cell adhesion molecule, epithelial cadherin, human epithelial receptor 2, etc.) of CTCs from blood cells, have emerged.^[^ ^8^, ^9^, ^10^, ^11^, ^12^, ^13^ ^]^ However, there are still some great challenges in CTCs isolation due to the inherent properties of CTCs: 1) scarcity: the number of CTCs in the bloodstream is extremely rare in contrast to blood cells (approximately 1 CTC per billion background cells). 2) Heterogeneity: epithelial‐specific markers (epithelial cell adhesion molecule, E‐cadherin, etc.) are selectively partially or completely downregulated during the metastatic process through the epithelial‐to‐mesenchymal transition.^[^ ^14^, ^15^, ^16^, ^17^, ^18^ ^]^ 3) Vulnerability: the cell viability of captured CTCs is susceptible to traditional enzyme degradation, mechanical lift‐off methods, and conventional three‐color immunoﬂuorescence identification, seriously impeding further downstream analysis.^[^ ^19^ ^]^ Therefore, developing a multifunctional CTCs isolation platform integrating high sensitivity, broad‐spectrum capture, in situ fluorescent identification, and cell‐friendly release is urgently desired.

To date, numerous techniques have been developed to address the abovementioned limitations. Among them, immunomagnetic separation strategy, in which magnetic nanoparticles are conjugated with specific antibodies as targeting ligands, has attracted extensive attentions due to its simplicity, rapidity, and specificity.^[^ ^20^ ^]^ Tannic acid (TA) is a widespread phenolic compound which occurs in various plants such as vegetables, olives, cacao, and others. Benefiting from the high content of catechol/pyrogallol groups, TA shows superior adherence to various substrates. Recently, it is found that there are strong hydrogen bonding and hydrophobic interactions between galloyl group‐containing TA and the glycocalyx structure of tumor cells.^[^ ^21^, ^22^ ^]^ And TA exhibits excellent resistance to the nonspecific adhesion of blood cells, making it an ideal candidate for broad‐spectrum capture of heterogeneous CTCs.^[^ ^23^, ^24^ ^]^ In addition to efficient capture, gentle release of the captured cancer cells with high viabilities is essential for downstream analysis. During tumor progression, indefinitely proliferative cancer cells mainly adopt glycolysis to meet their highly bioenergetic demands, resulting in the secretion of large amounts of lactic acid and causing acidification of the tumor microenvironment.^[^ ^25^ ^]^ In addition, it is reported that the speed of ATP production from glycolysis in cancer cells is approximately 100‐fold faster than that from oxidative phosphorylation in normal cells.^[^ ^26^ ^]^ Thus, we hypothesized that cancer cells have better tolerance to low pH and high‐level ATP, which means that cell‐friendly CTC release may be achieved in a pH/ATP‐responsive manner. Recently, zeolitic imidazolate frameworks (ZIF‐8) have attracted tremendous interests as promising drug carriers due to their unique merits, such as high porosity, large surface area, and tunable structure. It is noteworthy that ZIF‐8 has excellent pH/ATP‐responsiveness as a result of the protonation of 2‐methylimidazole linkers and the competitive Zn^2+^‐ATP coordination. Moreover, multiple fluorochromes and biomacromolecules can be encapsulated into ZIF‐8 by a simple biomimetic mineralization process. Inspired by these characteristics, ZIF‐8 appears to be an ideal vector in encapsulating imaging agents and disassembling under pH/ATP stimuli.^[^ ^27^, ^28^, ^29^, ^30^, ^31^, ^32^, ^33^ ^]^

**Scheme 1:**
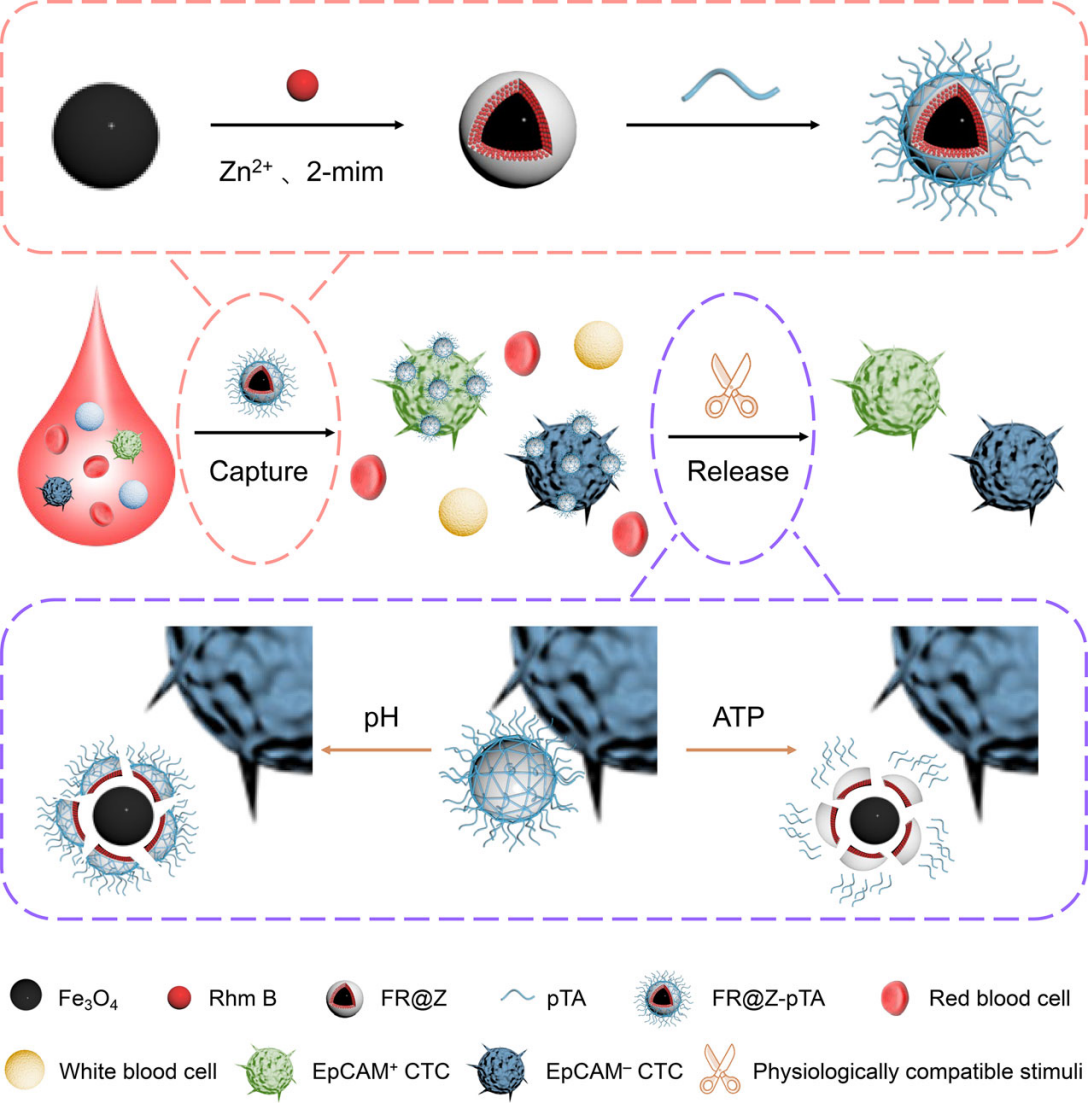
Schematic illustration showing the preparation of fluorescent‐magnetic FR@Z‐pTA NPs for broad‐spectrum capture and pH/ATP‐responsive release of heterogeneous CTCs from peripheral blood.

Herein, we developed a multifunctional platform based on pH/ATP‐responsive fluorescent‐magnetic Fe_3_O_4_/Rhm B@ZIF‐8‐pTA nanoparticles (FR@Z‐pTA NPs) for efficiently isolating heterogeneous CTCs (**Scheme** **1**). FR@Z‐pTA NPs were prepared by successively constructing Rhm B‐encapsulated ZIF‐8 shell and poly(tannic acid) (pTA) coating on the surface of superparamagnetic Fe_3_O_4_ nanoparticles through one‐step self‐assembly method and enzyme‐catalyzed oxidative polymerization. And the unique advantages of this platform are as follows: 1) Heterogeneous CTCs could be effectively captured by FR@Z‐pTA NPs due to the good affinity between pTA coating and proline‐rich proteins overexpressed on the surface of cancer cells. 2) Rhm B with high fluorescence quantum yield can be used for in situ identification of the captured cells, a procedure free of CTCs loss, and tedious procedures from additional identification. 3) The captured cells could be gently released under cell‐friendly stimuli (pH and ATP) due to the pH/ATP‐responsive degradation property of FR@Z‐pTA NPs, thus maintaining high cell viability for subsequent molecular analysis. As a result, we believe that the developed FR@Z‐pTA NPs integrating high sensitivity, broad‐spectrum capture, in situ fluorescent identification, and cell‐friendly release show great potential for sensitive detection of heterogeneous CTCs from clinical blood samples of cancer patients.

## 2 Results and Discussion

### 2.1 Characterization of the Nanoparticles

**Figure 1:**
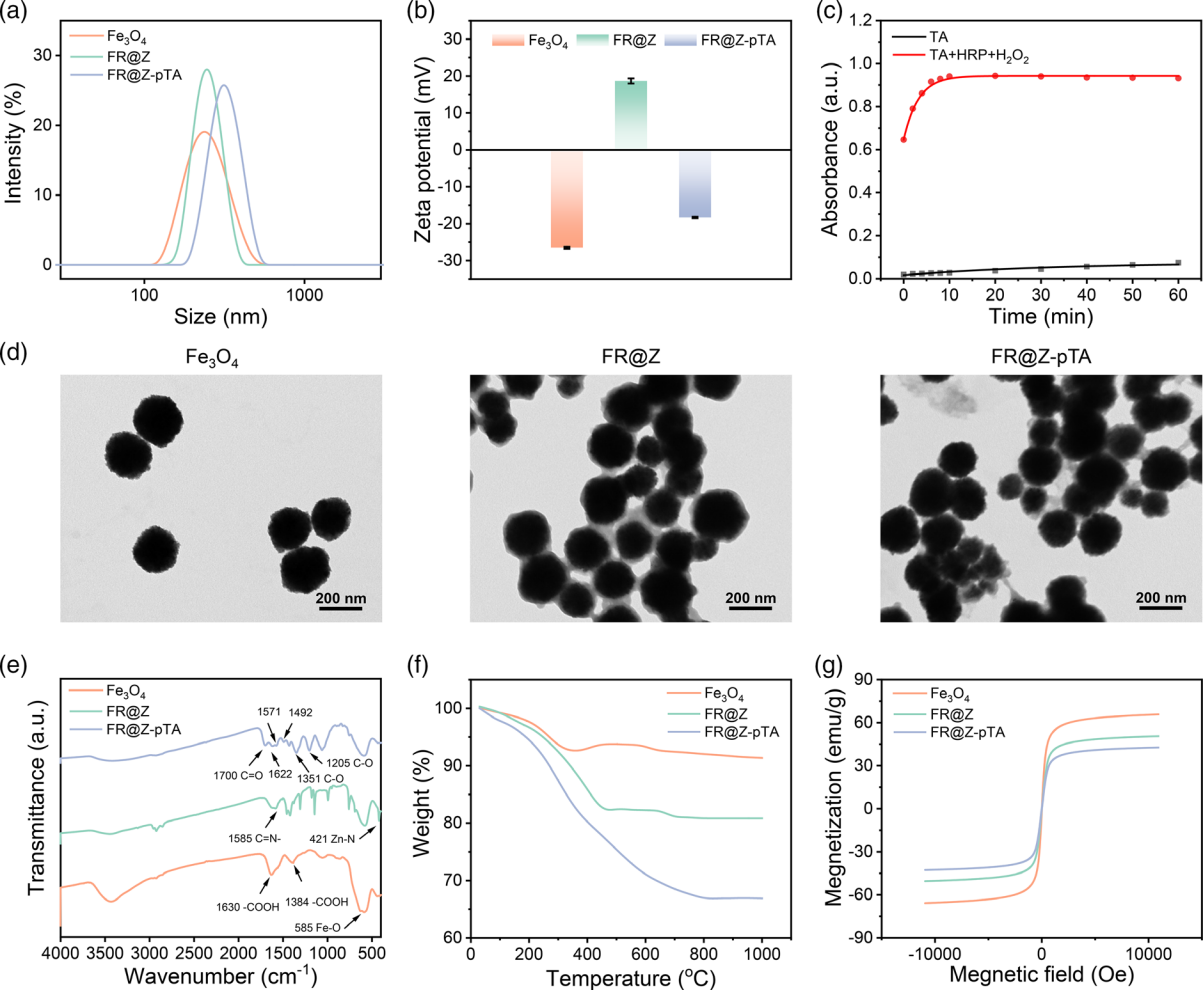
Characterization of different nanoparticles. a) Hydrodynamic size and b) zeta potential of different nanoparticles. c) UV–vis absorbance at 430 nm during TA polymerization at different time points. d) Transmission electron microscopy (TEM) images, e) FT‐IR spectra, f) TGA analysis, and g) magnetic hysteresis curves of different nanoparticles.

Fe_3_O_4_ NPs were prepared using a one‐step solvothermal method, and then the in situ grown of Rhm B‐encapsulated ZIF‐8 shell and pTA coating were successively constructed on Fe_3_O_4_ surface using one‐step self‐assembly approach and enzyme‐catalyzed oxidative polymerization method to obtain FR@Z‐pTA NPs. After the growth of ZIF‐8 shell and modification with pTA coating, the hydrodynamic particle size of nanoparticles increased from 232.1 to 246.4 nm and 313.6 nm (**Figure** **1**a), accompanied by obvious surface potential changes from −26.5 to 18.7 mV and −18.3 mV (Figure 1b). Enzyme‐catalyzed oxidative polymerization process of TA was monitored by UV–vis. As shown in Figure 1c, the absorbance of the TA + HRP + H_2_O_2_ reaction solution at 430 nm increased rapidly and reached a plateau after 10 min, while the TA solution only yielded a slight increase in absorbance after 60 min. This was due to that HRP catalyzed the oxidation of catechol in TA molecules to form quinone groups, which could in turn initiate polymerization of other TA molecules.^[^ ^34^ ^]^ As shown in Figure 1d, FR@Z NPs showed a distinct core–shell structure compared with the solid spherical structure of Fe_3_O_4_ NPs. And a light colored lamellar layer could be observed on the surface of FR@Z‐pTA NPs. These results demonstrated ZIF‐8 shell and pTA coating were successfully constructed on the surface of Fe_3_O_4_ NPs. Figure 1e shows that new absorption peaks at 1585 and 421 cm^−1^ originating from stretching vibrations of C=N– and Zn–N appeared in the Fourier transform infrared (FT‐IR) spectrum of FR@Z NPs in comparison to Fe_3_O_4_ NPs,^[^ ^35^, ^36^, ^37^ ^]^ indicating the successful growth of ZIF‐8 shell. pTA coating was further evidenced by the absorption peaks of C=O (1700 cm^−1^), benzene ring skeleton stretching vibrations (1622, 1571 and 1492 cm^−1^), and C=O (1351 and 1205 cm^−1^).^[^ ^38^ ^]^ The contents of ZIF‐8 shell and pTA coating were determined by thermogravimetric analysis (TGA) analysis, which revealed two areas of weight loss in the range of 30–350 and 350–1000 °C, respectively (Figure 1f). The former was caused by the residues of moisture and impurities, while the latter mainly resulted from the decomposition of the Rhm B‐containing ZIF‐8 shell and pTA coating. Through a comparison of the TGA curves of different nanoparticles, the contents of Fe_3_O_4_, Rhm B@ZIF‐8 and pTA were calculated to be 75.5%, 10.6%, and 13.9%, respectively. In addition, FR@Z‐pTA NPs showed good superparamagnetic property with a high saturation magnetization value of 42.7 emu g^−1^ (Figure 1g). Besides, FR@Z‐pTA NPs were quickly enriched within 3 min when exposed to an applied magnetic field and redispersed well again by gently shaking after withdrawing the magnet (Figure S1, Supporting Information). Moreover, FR@Z‐pTA NPs showed bright red fluorescence (Figure S2, Supporting Information). All these results demonstrated FR@Z‐pTA NPs exhibited excellent magnetic responsiveness and fluorescence property, which could lay a good foundation for the rapid separation and in situ fluorescent labeling of CTCs.

### 2.2 CTCs Capture and Identification Performance

**Figure 2:**
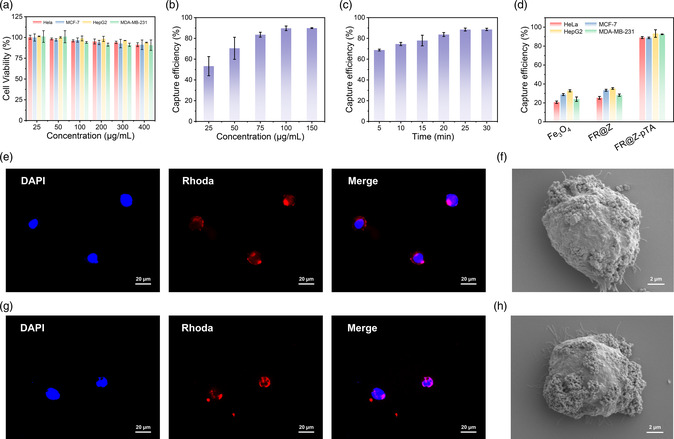
CTC‐capture and in situ fluorescent labeling performance of FR@Z‐pTA NPs. a) The cytocompatibility of FR@Z‐pTA NPs to different cancer cells. b,c) Capture efficiencies of HeLa cells by different b) concentration of FR@Z‐pTA NPs (b) and incubation durations (c). d) Capture efficiencies of different types of cancer cells by various nanoparticles. e–h) Typical fluorescence and SEM images of HeLa cells (e,f ) and HepG2 cells (g,h) captured by FR@Z‐pTA NPs.

EpCAM‐positive cells (MCF‐7, HepG2) and EpCAM‐negative cells (HeLa, MDA‐MB‐231) were used as model cells to check potential capture performance of FR@Z‐pTA NPs. First, the cytocompatibility of FR@Z‐pTA NPs was evaluated. As shown in **Figure** **2**a, all kinds of cancer cells showed high cell viability (>90%) in the tested concentration range (25–400 μg mL^−1^), indicating the good cytocompatibility of FR@Z‐pTA NPs. Then the capture performance of FR@Z‐pTA NPs toward cancer cells was systematically investigated. The microscope images showed that the surface of HeLa cells was fully covered with nanoparticles, indicating that FR@Z‐pTA NPs had good recognition and capture ability toward cancer cells (Figure S3, Supporting information). Subsequently, the concentration of FR@Z‐pTA NPs and incubation time for CTCs capture were optimized. The results showed that the capture efficiency gradually increased and reached 89.7% with increasing the concentration of FR@Z‐pTA NPs in the range of 0–100 μg mL^−1^, and remained constant afterward (Figure 2b). And the capture efficiency increased to a maximum with a incubation time of 25 min during the range of 5–30 min (Figure 2c). Subsequently, the capture performance of FR@Z‐pTA NPs toward heterogeneous cancer cells was further investigated. As shown in Figure 2d, FR@Z‐pTA NPs showed excellent capture efficiency (>88.7%) for different kinds of cancer cells under the optimal conditions. In contrast, Fe_3_O_4_ NPs and FR@Z NPs showed a lower capture efficiency (<40%) for cancer cells, which mainly resulted from nonspecific adsorption of nanoparticles on the cell surface. All these results demonstrated that FR@Z‐pTA NPs could effectively achieve broad‐spectrum capture of CTCs independent of their EpCAM expression levels. Interestingly, FR@Z‐pTA NPs bound onto the surface of both HeLa and HepG2 cells exhibited bright red fluorescence, indicating their good in situ fluorescent labeling property for the captured cells (Figure 2e,g). And the capture performance of FR@Z‐pTA NPs toward cancer cells could be further verified by scanning electron microscopy (SEM) images, which showed that plentiful nanoparticles bound onto the surface of captured cells. The efficiency and purity of CTCs are susceptible to nonspecific adhesion of white blood cells (WBCs) on the capture substrate. Therefore, the resistance of FR@Z‐pTA NPs to WBCs nonspecific adhesion was tested. It was shown that the anti‐WBC‐adhesion efficiency of FR@Z‐pTA NPs (86.9%) was much higher than that of FR@Z NPs (54.0%), demonstrating that pTA coating could effectively resist the nonspecific adhesion of WBCs (Figure S4, Supporting Information). The excellent resistance to WBCs adhesion is mainly attributed to the rich galloyl groups of TA, which could effectively downregulate the level of proinflammatory cytokines and inhibit the expression of leukocyte adhesion molecules.^[^ ^21^, ^39^ ^]^

**Figure 3:**
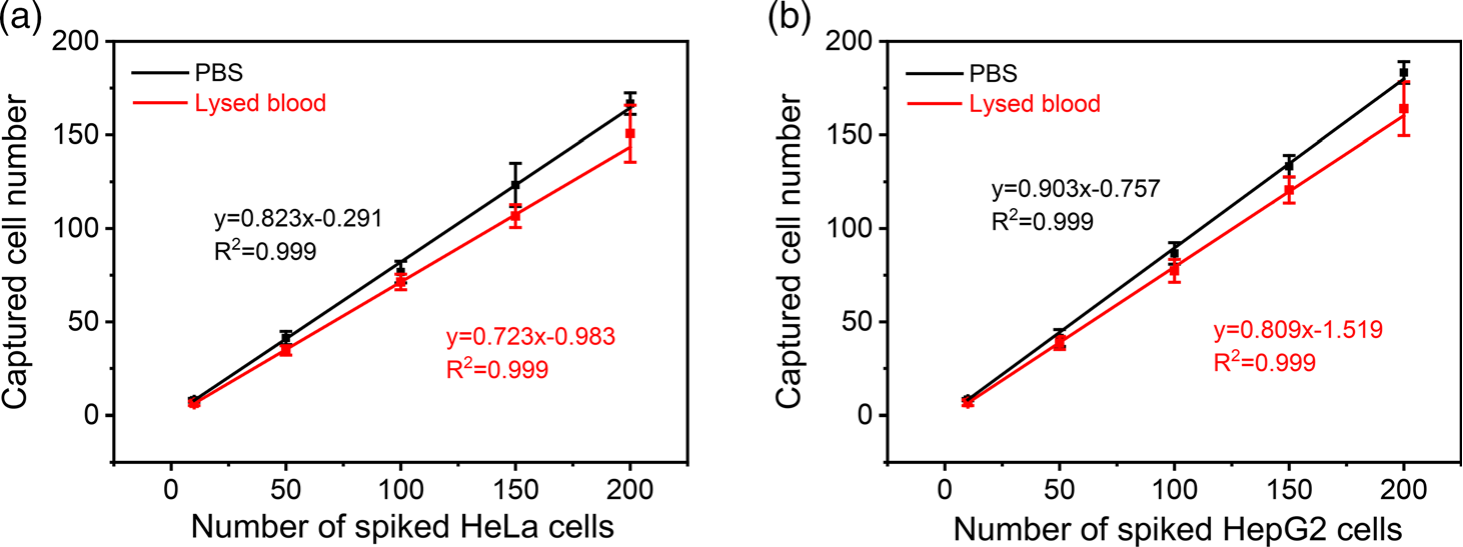
Detection sensitivity of FR@Z‐pTA NPs toward low‐concentration cancer cells in phosphate buffer saline (PBS) solution and lysed blood samples. a) Capture efficiencies of FR@Z‐pTA NPs toward HeLa cells spiked in PBS solution and lysed blood samples with low concentration (10–200 cells mL^−1^). b) Capture efficiencies of FR@Z‐pTA NPs toward HepG2 cells spiked in PBS solution and lysed blood samples with low concentration (10–200 cells mL^−1^).

To investigate the detection sensitivity of FR@Z‐pTA NPs toward different cancer cells, FR@Z‐pTA NPs were applied to detect HeLa and HepG2 cells in artificial and simulated samples containing 10–200 cells mL^−1^. As shown in **Figure** **3**a, the average capture efficiencies of FR@Z‐pTA NPs to HeLa cells in artificial and simulated samples reached 82.3% and 72.3%, respectively. And a good linear relationship prevailed between the number of captured cells and spiked cells. Similar results were observed in artificial and simulated samples spiked with HepG2 cells, whose capture efficiencies were 90.3% and 80.9%, respectively (Figure 3b). All these results demonstrated that FR@Z‐pTA NPs could recognize and capture rare heterogeneous CTCs from complex physiological environment with excellent detection sensitivity.

### 2.3 Stimuli‐Responsive CTCs Release Performance and Cell Viability Assay

**Figure 4:**
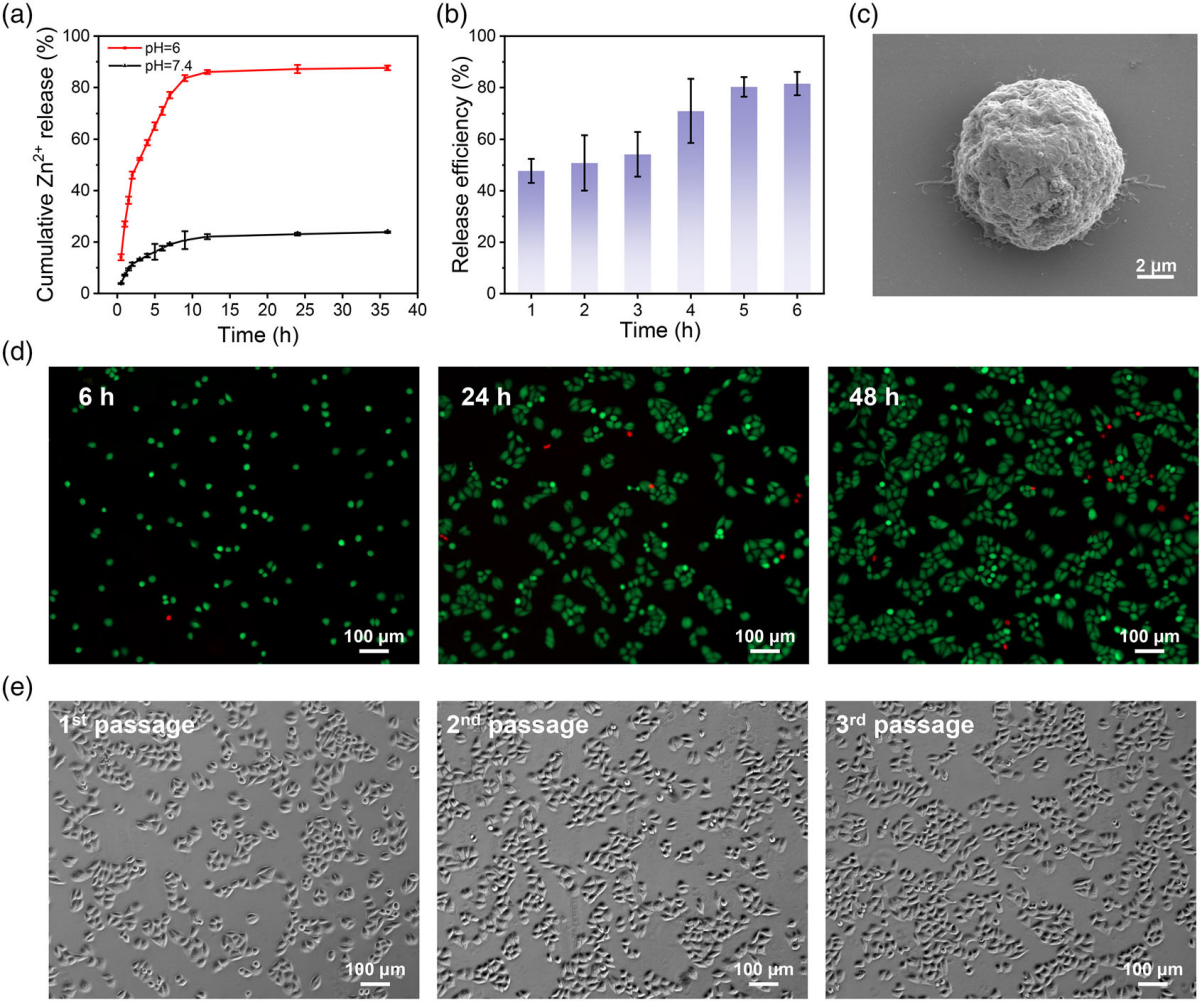
pH‐responsive CTCs release and functional studies of the recovered cells. a) Time‐dependent release of Zn^2+^ from FR@Z‐pTA NPs at various time points in PBS with different pH. b) Release efficiency of captured HeLa cells after culture in serum‐free medium (pH = 6) for different times. c) Typical SEM image of released HeLa cell from FR@Z‐pTA NPs. d) Fluorescence images showing released HeLa cells after live/dead staining. e) Microscopy images showing released cells reculture for different passages.

Beyond CTCs capture and identification, it is important to achieve efficient and cell‐friendly release of the captured cells, which is crucial for further downstream analysis. It was reported that ZIF‐8 exhibited pH‐responsiveness as the 2‐methylimidazole linkers were protonated under acidic condition, thus disrupting Zn^2+^‐imidazole coordination interactions and decomposing the ZIF‐8 structure.^[^ ^40^ ^]^ Therefore, the captured CTCs on FR@Z‐pTA NPs could be released under a slightly acidic environment. To verify this, FR@Z‐pTA NPs were incubated in acidic PBS (pH = 6), followed by monitoring Zn^2+^ release. **Figure** **4**a shows that about 15% Zn^2+^ release was observed in neutral PBS solution (pH = 7.4) after incubation for 5 h. This was mainly caused by the affinity of phosphate ions toward Zn^2+^, thus causing slight decomposition of the sod‐Zn(mim)_2_ crystalline network.^[^ ^41^, ^42^ ^]^ In contrast, the cumulative release of Zn^2+^ reached more than 60% (pH = 6) after 5 h of incubation, demonstrating the pH‐responsive disintegration of the ZIF‐8 shell. Subsequently, the pH‐responsive release behavior of captured CTCs and functional studies of the recovered cells were systematically studied. As shown in Figure 4b, the release efficiency of CTCs reached 80.3% under the above conditions, and nanoparticles were hardly observed on the released cell with an whole morphology (Figure 4c). In addition, the cell viability and proliferative capability of recovered cells were further investigated. The results demonstrated that the recultured cells showed high viability (>90%) and good proliferation ability (Figure 4d,e and S5, Supporting Information). These results demonstrated that the pH‐responsive properties of FR@Z‐pTA NPs provided a convenient and gentle manner for the cell‐friendly release of captured CTCs.

**Figure 5:**
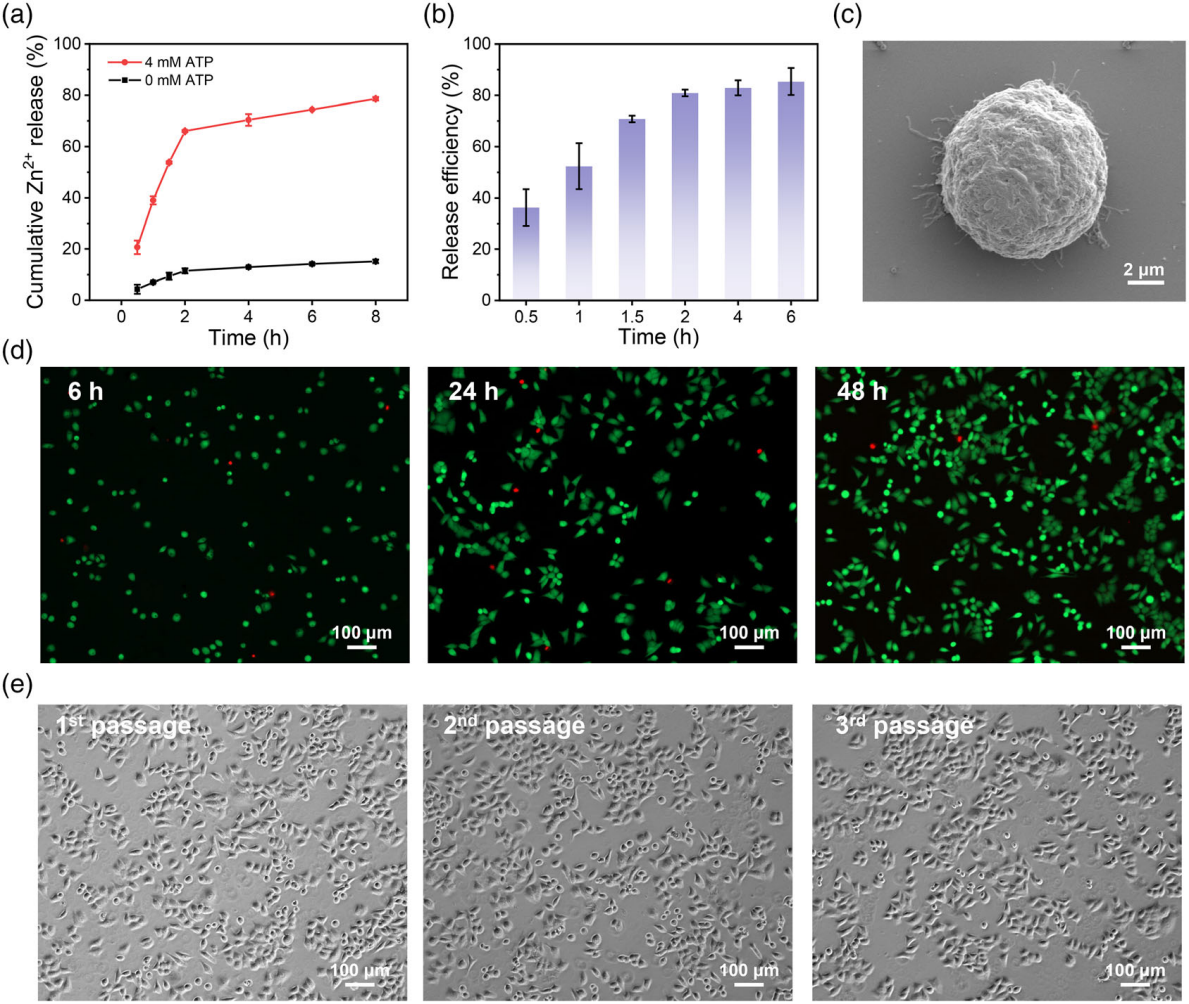
ATP‐responsive CTCs release and functional studies of the recovered cells. a) Time‐dependent release of Zn^2+^ from FR@Z‐pTA NPs at various time points in the absence and presence of ATP. b) Release efficiency of captured HeLa cells after culture in serum‐free medium containing 4 × 10^−3^ m ATP for different times. c) Typical SEM image of released HeLa cell from FR@Z‐pTA NPs. d) Fluorescence images showing released HeLa cells after live/dead staining. e) Microscopy images showing released cells reculture for different passages.

Besides pH, ZIF‐8 is also found to be decomposed by subcellular ATP which could form stronger Zn–triphosphate coordination with Zn^2+^ than the Zn–imidazole bond.^[^ ^43^ ^]^ In addition, ATP exhibited higher binding affinity to Zn^2+^ through Zn–triphosphate coordination than the Zn–phenolic coordination.^[^ ^44^ ^]^ Therefore, it is anticipated that ATP may result in the degradation of ZIF‐8 shell layer and shedding of pTA coating. To verify our hypothesis, the ATP‐responsive degradation behavior of FR@Z‐pTA NPs was investigated by atomic absorption spectrophotometer (AAS) (**Figure** **5**a). In the presence of 4 mm ATP, FR@Z‐pTA NPs showed a rapid initial release of Zn^2+^ (65.9%) within 2 h followed by a slow release. In contrast, about 10% Zn^2+^ was released from the FR@Z‐pTA NPs after 8 h in the absence of ATP. These results showed that FR@Z‐pTA NPs exhibited sensitive responsiveness to ATP due to its strong coordination ability with Zn^2+^. Subsequently, the ATP‐responsive release of cancer cells was studied. As shown in Figure 5b, the cell release efficiency reached 80.9% after incubation for 2 h and hardly changed with extending time, which was consistent with the trend of Zn^2+^ release. This could also be verified from typical SEM images of the released cells with a whole morphology with few adhered nanoparticles (Figure 5c). Next, the cellular viability and proliferative capacity were studied. The live/dead straining results showed that only a few dead cells labeled with red fluorescent PI were observed after reculturing for different times (Figure 5d). And the cell viability of released CTCs was over 95% after reculturing for different time (Figure S6, Supporting Information). Moreover, the cells grew well and retained good proliferation ability after several passages (Figure 5e). These results demonstrated that the ATP‐responsive properties of FR@Z‐pTA NPs could achieve effective and cell‐friendly release of CTCs without damaging their viability and proliferation capacity. In contrast to pH, ATP stimulus is superior because it could achieve a faster cell release (2 vs 5 h) and maintain higher cell viability (95% vs 90%).

### 2.4 CTCs Detection from Clinical Patient Blood Samples

**Figure 6:**
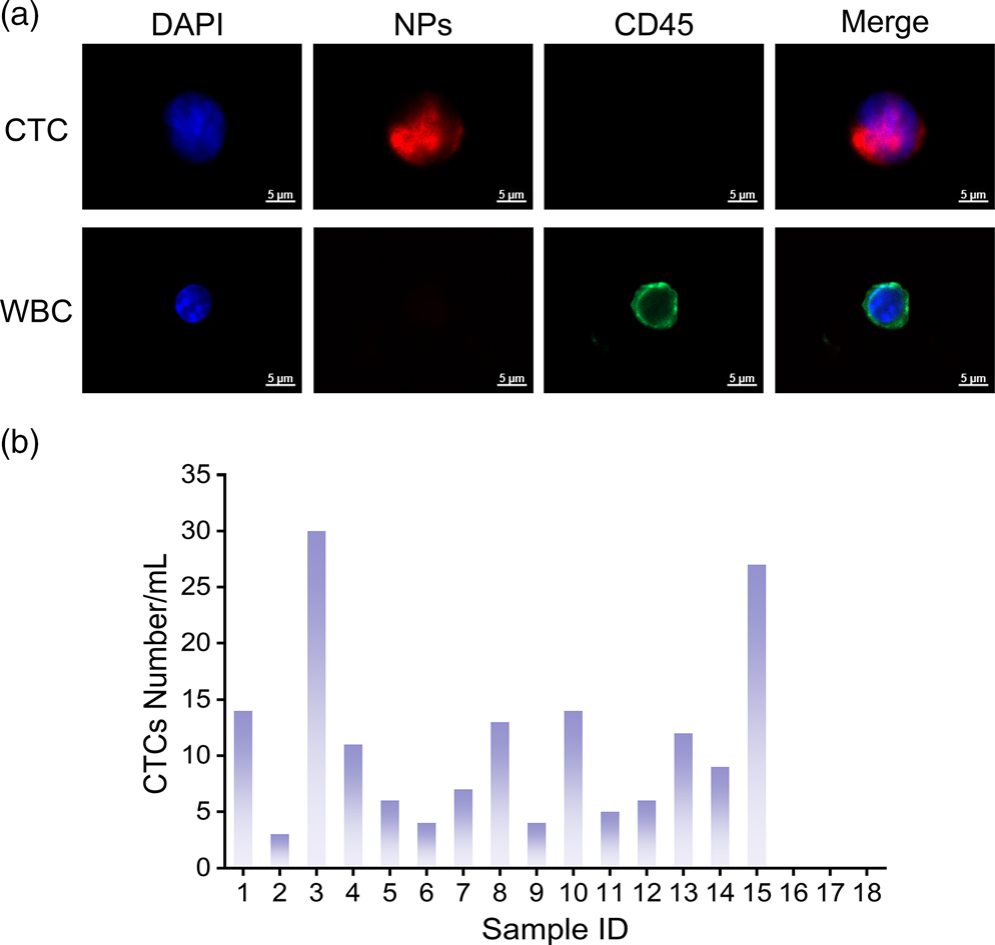
Detection of CTCs using FR@Z‐pTA NPs from clinical patients’ blood samples. a) Representative fluorescence images of captured single CTC and WBC. b) Numbers of isolated CTCs from blood samples of cancer patients and healthy donors.

To evaluate the feasibility of FR@Z‐pTA NPs for clinical samples, CTCs detection was performed using the blood samples of 15 cancer patients (colon, ovarian, esophageal, lung, stomach, and lymphoma) and 3 healthy donors. The isolated cells were identified by immunocytochemistry, including DAPI for labeling cell nucleus, the fluorescent FR@Z‐pTA NPs for labeling tumor cells, and Alexa Fluor 488‐anti‐CD45 for labeling CD45 antigen overexpressed on WBCs surface.^[^ ^45^ ^]^ As shown in **Figure** **6**a, cells showing DAPI+/NPs+/CD45− signals were identified as CTCs and cells with DAPI+/NPs‐/CD45+ signals were defined as WBCs. The results of CTCs enumeration showed that 3–30 CTCs mL^−1^ were detected from patients’ blood samples, while no CTCs were found for the healthy donors (Figure 6b and Table S1, Supporting Information). These results demonstrated FR@Z‐pTA NPs could effectively detect rare CTCs from clinical patients’ blood, holding great promise for application in clinical setting.

## 3 Conclusion

We have demonstrated a facile strategy for the fabrication of a multifunctional platform (based on FR@Z‐pTA NPs) for CTCs isolation. FR@Z‐pTA NPs showed high capture efficiency (>88%) toward both EpCAM‐positive cells (MCF‐7, HepG2) and EpCAM‐negative cells (HeLa, MDA‐MB‐231). In addition, FR@Z‐pTA NPs could effectively resist the nonspecific adhesion of 86.9% WBCs and detect rare spiked cancer cells in simulated blood samples (10–200 cells mL^−1^). Moreover, the captured cells not only were simultaneously fluorescent‐labeled by FR@Z‐pTA NPs for in situ identification, but also could be gently released with high efficiency (>80%) and viability (>90%) under cell‐friendly stimuli (pH and ATP). More importantly, FR@Z‐pTA NPs successfully achieved effective detection of CTCs (3–30 CTCs mL^−1^) from 15 clinical blood samples with six types of cancer. This multifunctional platform integrating high sensitivity, broad‐spectrum capture, in situ fluorescent identification, and cell‐friendly release is expected to be a promising way to realize efficient isolation of heterogeneous CTCs, showing great potential for sensitive detection of heterogeneous CTCs from clinical blood samples of cancer patients.

## 4 Experimental Section

The experimental details are provided in the Supporting Information. All experiments were performed in accordance with the tenets of the Declaration of Helsinki, and approved by the the Ethics Committee of Sichuan Academy of Medical Sciences and Sichuan Provincial People's Hospital (2021241). Informed consent was obtained from the human participants of this study.

## Conflict of Interest

The authors declare no conflict of interest.

## Supporting information

Supplementary Material

## Data Availability

The data that support the findings of this study are available from the corresponding author upon reasonable request.
