# Supplementary material for: Efficient Isolation and In Situ Identification of Viable Circulating Tumor Cells Using Dual‐Responsive Fluorescent‐Magnetic Nanoparticles
Source: Small Sci. 2022 Oct 27;2(12):2200061. doi: 10.1002/smsc.202200061 (PMC11936015; doi:10.1002/smsc.202200061)
Supplement: Supplementary file 1 — Supplementary Material [file SMSC-2-2200061-s001.pdf]

Supporting Information

Efficient Isolation and in Situ Identification of Viable Circulating Tumor Cells Using Dual-Responsive Fluorescent-Magnetic Nanoparticles

Yuwei Zhou, Xiaoshan Wang, Zhouying Luo, Xia Liu, Jianwen Hou and Shaobing Zhou**

Y. Zhou, Z. Luo, Prof. J. Hou, Prof. S. Zhou

Key Laboratory of Advanced Technologies of Materials, Ministry of Education

School of Materials Science and Engineering, Southwest Jiaotong University

Chengdu 610031, China

E-mail: houjianwen@swjtu.edu.cn; shaobingzhou@swjtu.edu.cn

Prof. X. Wang

Cancer Center, Hospital of The University of Electronic Science and Technology of China and Sichuan Provincial People's Hospital

School of Medicine, University of Electronic Science and Technology of China, Chengdu 610072, China

Prof. X. Liu

School of Life Science and Engineering, Southwest Jiaotong University, Chengdu 610031, China

1. Experimental Section

1.1 Chemicals

Iron chloride hexahydrate ($FeCl_3$ $6H_2O$), zinc nitrate hexahydrate ($ZnNO_3$ $6H_2O$) and peroxidase from horseradish (HRP) were purchased from Aladdin Reagent Co., Ltd. (Shanghai, China). Sodium citrate dehydrate (Na_3Cit $2H_2O$)and sodium acetate (NaAc) were purchased from Chengdu Kelong Chemical Reagent Company. Ethylene glycol (EG), 2-methylimidazole (2-mim), adenosine triphosphate (ATP), tannic acid (TA) and rhodamine B (Rhm B) were purchased from Adamas-beta (Shanghai, China). 4′,6-diamidine-2′-phenylindole dihydrochloride (DAPI), propidium iodide (PI), calcein AM and Alamar Blue were purchased from Sigma-Aldrich (USA). ACK Lysis Buffer was purchased from Solarbio Life Sciences Co. Ltd (Beijing, China). Alexa Fluor 488-labeled anti-CD45 was purchased from Abcam (Shanghai, China).

1.2 Characterization

The size distribution and zeta-potential of nanoparticles were measured by a dynamic light scattering (DLS) analyzer (Nano-ZS90, Malvern, U.K.). The morphology of the nanoparticles was characterized by transmission electron microscopy (JEM-1400PLUS, JEOL, Japan). The chemical composition of nanoparticles was characterized by fourier transform infrared spectroscopy (Nicolet iS50, Thermo Fisher Scientific, America). Thermogravimetric analysis (TGA) was conducted on a thermal analyzer (STA 449C, Netzsch, Germany). The primary thermograms were recorded in the temperature range of ambient to 1000 ℃ at a heating rate of 10 ℃ min^{-1} under a nitrogen atmosphere. Magnetic hysteresis loops were measured with a vibrating sample magnetometer (VSM, 7404, Lakeshore, America). The cell morphologies were observed with a fluorescence microscope (Axio Observer, Zeiss, Germany) and field emission scanning electron microscope (FE-SEM, JSM-7800F, JEOL, Japan). The cumulative release of zinc ions was measured by atomic absorption spectrophotometer (AAS, TAS-990F, PERSEE, China).

1.3 Synthesis of Fe_3O_4 NPs

Fe_3O_4 NPs were synthesized by a solvothermal method.[1,2] Firstly, $FeCl_3 \cdot 6H_2O$ (1.14 g) and $Na_3Cit \cdot 2H_2O$ (0.74 g) were dissolved in 50 mL of EG and vigorously stirred at 45 ^oC for 30 min. Then NaAc (2.40 g) was added into the above solution and stirred for 1 h. The resulting solution was transferred to an autoclave sealed with polytetrafluoroethylene (PTFE) and heated at 200 ℃ for 10 h. The Fe_3O_4 NPs were separated by a magnet, washed three times with ethanol and deionized water, sealed and stored in a refrigerator at 4 ℃.

1.4 Preparation of FR@Z NPs

FR@Z NPs were prepared by a one-pot method.[3] 2-mim (820 mg) was dissolved in 25 mL of methanol, and of Fe_3O_4 NPs (10 mg) were added and sonicated for 15 min to achieve uniform dispersion of the nanoparticles. Then 5 mL of methanol solution containing Rhm B (2 mg) and $ZnNO_3 \cdot 6H_2O$ (100 mg) was slowly added under light-proof conditions. After 1 h of reaction, FR@Z NPs were separated by a magnet, washed three times with methanol, sealed and stored in a refrigerator at 4 ℃ under light-proof conditions.

1.5 Preparation of FR@Z-pTA NPs

pTA coating was modified by enzyme-catalyzed oxidative polymerization method. Under light-proof conditions, FR@Z NPs (10 mg) and TA (10 mg) were dispersed in 15 mL and 5 mL of Tris-HCl (10 mM, pH=8.5), respectively. Then the above solutions were mixed by vortexing for 30 min at room temperature. Followingly, 100 μL of H_2O_2 (1 M) and 50 μL of HRP (100 μM) were added into the mixed solution and reacted in a shaker at 37°C for 60 min. The obtained FR@Z-pTA NPs were separated by a magnet, washed three times with deionized water, sealed and stored in a refrigerator at 4 ℃ away from light.

1.6 Cell Culture and Blood Sample Processing

HeLa, MDA-MB-231, MCF-7 and HepG2 cells with different levels of EpCAM expression were used as model cells. HeLa, MDA-MB-231 and HepG2 cells were cultured in DMEM

medium supplemented with 10% FBS. MCF-7 cells were cultured in RPMI 1640 medium supplemented with 10% FBS.

Human blood samples were provided by the Academy of Medical Sciences and Sichuan Provincial People's Hospital (blood collection was performed according to the guidelines issued by the Ethical Committee of the Academy of Medical Sciences and Sichuan Provincial People's Hospital). Then the whole blood samples were treated with ACK lysis buffer according to the manufacturer's instructions.

1.7 Capture Efficiency of FR@Z-pTA NPs

Firstly, the cytocompatibility of FR@Z-pTA NPs was evaluated. FR@Z-pTA NPs with different concentrations (25-400 μg/mL) were incubated with four tumor cells (HeLa, MDA-MB-231, MCF-7 and HepG2 cells) for 24 h, and the activity of the cells was investigated by Alamar Blue method. To optimize the concentration of nanoparticles for cell capture, 1.5×10^5 HeLa cells were incubated with different concentrations of FR@Z-pTA NPs (25-150 μg/mL) in a shaker for 30 min at 37 ℃. Then the captured cells were collected by magnetic separation and then washed three times with PBS to remove uncaptured cells for counting by hemocytometer. The number of captured cells was obtained by subtracting the number of uncaptured cells from the number of added cells. The capture efficiency was defined as the ratio of the number of captured cells to the number of added cells. In addition, the effect of incubation time on capture efficiency was determined by incubating HeLa cells with FR@Z-pTA NPs under optimal concentrations for different times (1-30 min). Finally, FR@Z-pTA NPs were incubated with EpCAM-positive cells (HepG2 and MCF-7 cells) and EpCAM-negative cells (MDA-MB-231 cells) under optimal conditions to demonstrate their ability to broad-spectrum capture heterogeneous CTCs.

1.8 Rare-Cell Capture Performance of FR@Z-pTA NPs

For rare-cell capture studies, aritifical samples were prepared by adding different numbers of HeLa or HepG2 cells (10-200 cells) into 1 mL of PBS. Similarly, simulated samples were prepared by spiking different numbers of HeLa or HepG2 cells (10-200 cells) into 1 mL of processed blood. Following the above procedures, the rare-cell capture performance of FR@Z-pTA NPs was evaluated.

1.9 Release Efficiency and Viability of Captured CTCs

Firstly, the ATP- and pH-responsive degradation performances of FR@Z-pTA NPs in PBS with ATP=4 mM or pH=6 were examined by measuring the cumulative release of Zn^{2+} using AAS. Then, the captured cells were further recultured in high-glucose DMEM (ATP=4 mM or pH=6) for different time periods. After washing, all the released cells were collected in the supernatant and counted using a hemocytometer under inverted phase contrast microscope. The release efficiency was defined as the ratio of the number of released cells to the number of captured cells. Cell viability was tested by the live/dead staining method. The released cells were re-cultured for 6, 24 and 48 h, followed by staining with calcein AM and PI. Cells staining positive for calcein AM or PI were deemed alive or dead, respectively. Lastly, the released cells were cultured for 48 h and transferred to a new well plate for further incubation and subcultured when cells spread at the bottom of the plate.

1.10 CTCs Detection from Clinical Blood Samples

Peripheral blood samples were collected from 15 cancer patients with different cancer types and 3 healthy volunteers using blood collection tubes containing EDTA. The blood samples were treated with ACK lysis buffer and incubated with FR@Z-pTA NPs under optimal conditions for CTCs isolation. Isolated cells were fixed with 2.5% glutaraldehyde for 2 h, permeabilized with 0.1% Triton X-100 for 5 min and blocked with 1% BSA for 1 h. The cells were then incubated with Fluor 488-labeled anti-CD45 (20 μg/mL in PBS) overnight at 4 ℃. Finally, the captured cells were stained with DAPI (5 μg/mL in PBS) for 15 min and observed with a confocal microscope.

1.11 Ethical Approval

All experiments were performed in accordance with the tenets of the Declaration of Helsinki, and approved by the the Ethics Committee of Sichuan Academy of Medical Sciences and Sichuan Provincial People's Hospital (2021241). Informed consents were obtained from the human participants of this study.

2. Supplementary Figures

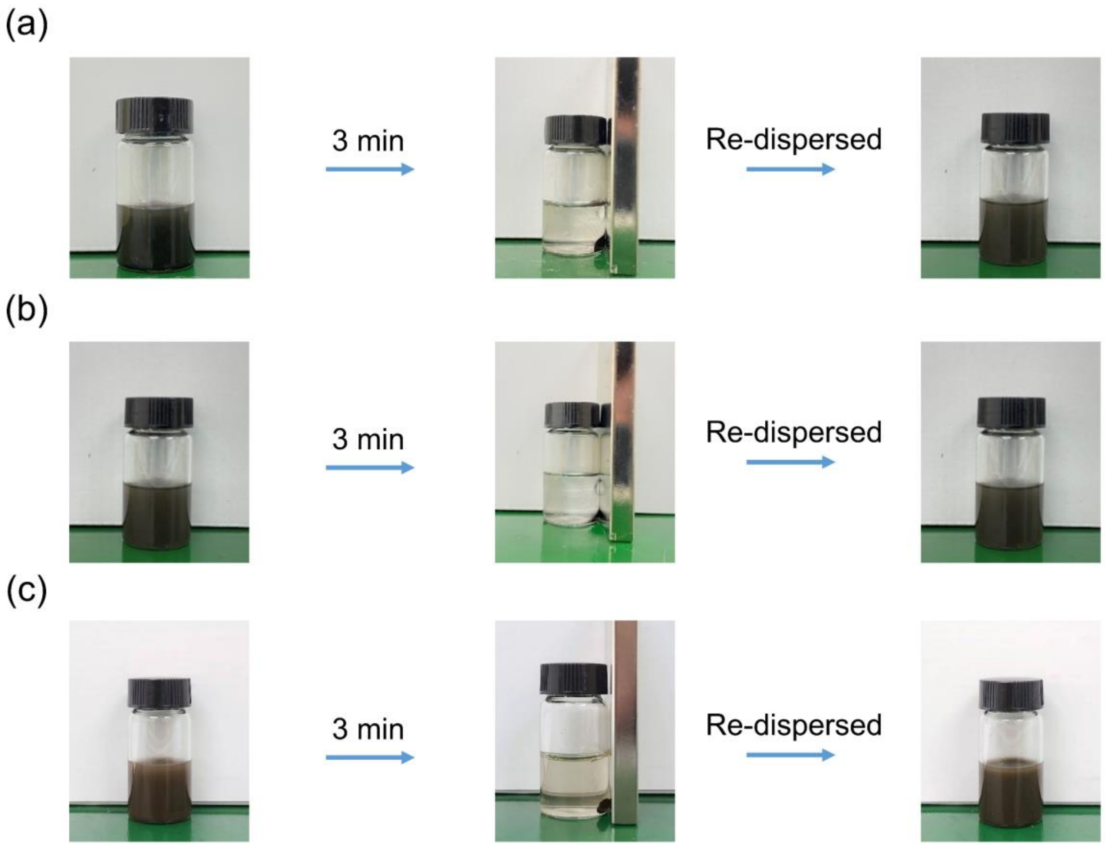

Figure S1. Typical images showing the magnetic responsiveness and dispersion properties of a) Fe_3O_4 NPs , b) FR@Z NPs, and c) FR@Z-pTA NPs.

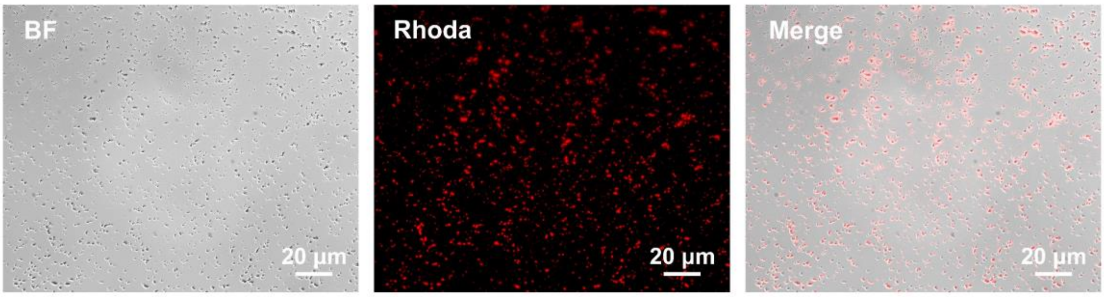

Figure S2. Bright field (BF), fluorescent, and merged images of FR@Z-pTA NPs.

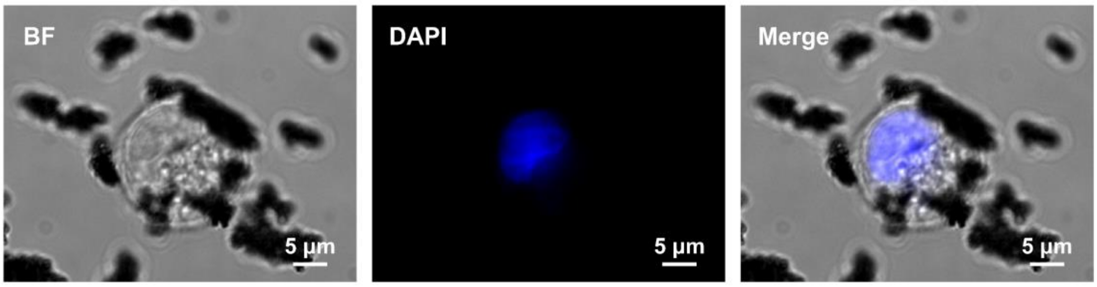

Figure S3. Bright field (BF), fluorescent, and merged images of HeLa cells captured by F@Z-pTA NPs.

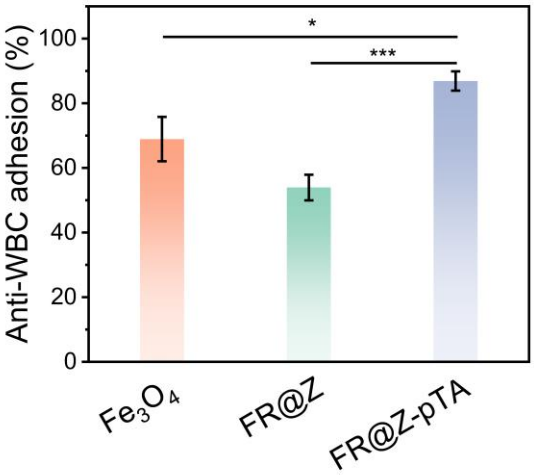

Figure S4. The resistance performance of different nanoparticles to nonspecific adhesion of WBCs. *$p < 0.05$, ***$P < 0.001$

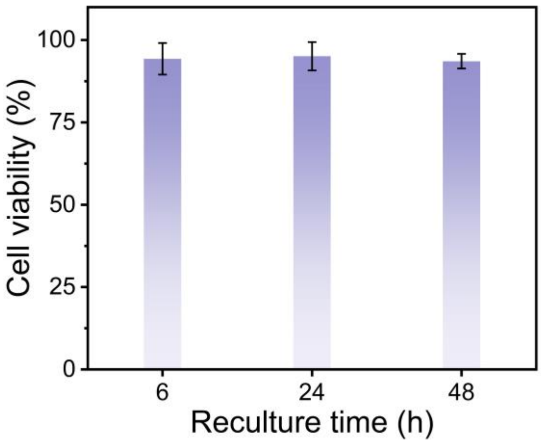

Figure S5. The cell viability of pH-responsive released CTCs after different reculture time.

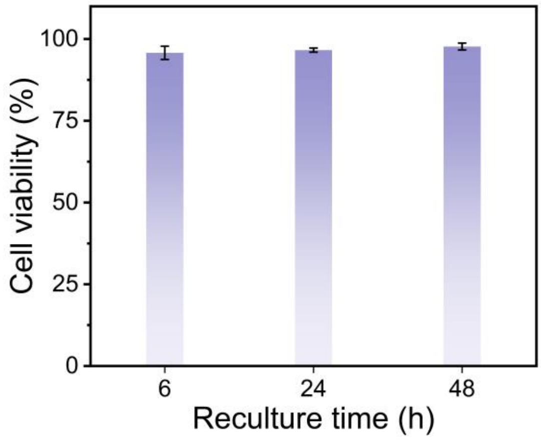

Figure S6. The cell viability of ATP-responsive released CTCs after different reculture time.

3. Supplementary Tables

Table S1. Basic information of cancer patients and healthy donors.

Diagnosis	Sample ID	Gender	Age	Clinical stage	CTC counts
Colon cancer	1	male	21	IV	14
Ovarian cancer	2	female	67	IV	3
	3	female	67	III	30
Esophagus cancer	4	male	52	IV	11
Lung cancer	5	female	50	IV	6
	6	female	52	IV	4
	7	male	62	limited	7
	8	male	72	extensive	13
	9	male	54	II	4
	10	male	55	III	14
	11	male	67	III	5
	12	female	46	IV	6
Gastric cancer	13	male	54	IV	12
	14	male	62	III	9
Lymph cancer	15	female	71	III	27
Healthy donor	16	male	25	N/A	0
	17	male	27	N/A	0
	18	male	33	N/A	0

4. References

[1] J. Liu, Z. Sun, Y. Deng, Y. Zou, C. Li, X. Guo, L. Xiong, Y. Gao, F. Li, D. Zhao, *Angew. Chem., Int. Ed.* **2009**, *48*, 5875.

[2] Y. Wang, G. Wei, X. Zhang, X. Huang, J. Zhao, X. Guo, S. Zhou, *Small* **2018**, *14*, 1702994.

[3] W. Xie, C. Gao, J. Li, *Renewable Energy* **2021**, *168*, 927.
